# Immune checkpoint inhibitors combined with concurrent chemoradiotherapy in locally advanced esophageal squamous cell carcinoma

**DOI:** 10.3389/fimmu.2024.1355198

**Published:** 2024-03-14

**Authors:** Jiang-Qiong Huang, Huan-Wei Liang, Yang Liu, Long Chen, Su Pei, Bin-Bin Yu, Xin-Bin Pan

**Affiliations:** Department of Radiation Oncology, Guangxi Medical University Cancer Hospital, Nanning, Guangxi, China

**Keywords:** esophageal squamous cell carcinoma, immune checkpoint inhibitors, concurrent chemoradiotherapy, survival, immunotherapy

## Abstract

**Purpose:**

This study aims to evaluate the efficacy of immune checkpoint inhibitors (ICIs) combined with concurrent chemoradiotherapy (CCRT) versus CCRT alone in patients with locally advanced esophageal squamous cell carcinoma.

**Materials and methods:**

This retrospective cohort study included patients diagnosed with locally advanced esophageal squamous cell carcinoma who received either CCRT alone or CCRT combined with ICIs from April 2019 to February 2023. The primary endpoint was progression-free survival (PFS), and the secondary endpoint was overall survival (OS).

**Results:**

A total of 101 patients were enrolled, with 58 undergoing CCRT alone and 43 receiving CCRT+ICI. The CCRT+ICI group demonstrated a higher complete response rate compared to the CCRT alone group (11.6% vs. 1.7%, P = 0.037). However, no significant difference was observed in 1-year PFS (58.9% vs. 55.2%; hazard ratio [HR] = 1.26, 95% confidence interval [CI]: 0.70-2.26; P = 0.445) or 1-year OS (70.8% vs. 75.9%; HR = 1.21, 95% CI: 0.58-2.53; P = 0.613) between CCRT+ICI and CCRT alone groups. The CCRT alone group experienced a higher incidence of leukopenia of any grade (93.1% vs. 76.7%, P = 0.039) but a lower incidence of pneumonitis of any grade (36.2% vs. 65.1%, P = 0.008).

**Conclusion:**

CCRT+ICI may not lead to improved survival outcomes compared to CCRT alone in patients with locally advanced esophageal squamous cell carcinoma. These findings indicate the need for further investigation into this treatment approach.

## Introduction

Esophageal cancer is recognized as the sixth leading cause of cancer-related mortality globally (1), with China accounting for over half of the newly diagnosed cases worldwide (2). Predominantly, squamous cell carcinoma represents about 90% of these cases in China (1). For patients with unresectable, locally advanced esophageal cancer, definitive concurrent chemoradiotherapy (CCRT) is the standard of care. Despite this, approximately 50% of patients still experience disease progression (3–5), leading to a 3-year overall survival (OS) rate that varies between 26.9% and 55.4% (3–7). This underscores the urgent need for more effective treatment strategies for this patient population.

In recent years, immune checkpoint inhibitors (ICIs) have gained prominence as a viable second-line treatment, offering improved survival outcomes in advanced esophageal cancer (8). Particularly noteworthy is the combination of ICIs with chemotherapy as a first-line treatment, which has been shown to be effective and safe in patients with stage IVB disease (9–15). Furthermore, integrating ICIs with CCRT has been reported to exhibit synergistic effects, enhancing survival benefits in patients with locally advanced disease (16–19). However, the relative efficacy and safety of CCRT combined with ICIs versus CCRT alone in this context remain to be fully elucidated. This study, therefore, aims to evaluate the efficacy and safety of CCRT+ICI in comparison to CCRT alone in patients with locally advanced esophageal squamous cell carcinoma.

## Materials and methods

### Patients

This retrospective cohort study included patients diagnosed with esophageal squamous cell carcinoma between April 2019 and February 2023. Inclusion criteria were (1): histopathologically confirmed esophageal squamous cell carcinoma (2), classification as locally advanced stage, and (3) treatment with definitive concurrent chemoradiotherapy (CCRT). Exclusion criteria included (1): presence of secondary malignancies (2), incomplete radiotherapy, and (3) prior receipt of neoadjuvant or adjuvant therapies.

Pre-treatment evaluations comprised physical examinations, standard hematological and biochemical tests, barium esophagograms, esophagogastroduodenoscopy with endoscopic ultrasound, contrast-enhanced cervical, thoracic, and abdominal computed tomography scans, brain magnetic resonance imaging, bone scans, electrocardiograms, echocardiograms, and pulmonary function tests. 18-fluorodeoxyglucose positron emission tomography/computed tomography was performed in cases where distant metastasis was suspected, and bronchoscopies were conducted if bronchial or tracheal invasion was suspected.

### Radiotherapy

All patients received intensity-modulated radiotherapy using 6-MV photons. The gross tumor volume (GTV) included the primary tumor (GTVp) and metastatic lymph nodes (GTVn), identified via endoscopy, contrast-enhanced thoracic computed tomography, and/or 18-fluorodeoxyglucose positron emission tomography/computed tomography. The primary clinical target volume (CTVp) included GTVp with an additional radial margin of 0.5-1.0 cm and longitudinal margins of 3 cm. The clinical target volume for metastatic lymph nodes (CTVn) was delineated based on involved field irradiation, excluding elective nodal irradiation (20, 21). The planning target volume (PTV) was defined by adding a margin of 0.5-1.0 cm to the CTV. Cone beam CT was used for daily verification during the first week of treatment, followed by weekly verification thereafter.

Radiotherapy was delivered five days per week, targeting a dose of either 60.0 Gy in 30 fractions or 50.4 Gy in 28 fractions. Dose constraints for organs at risk were set as follows (1): mean lung dose < 17 Gy, lung V20 < 30%, and lung V5 < 65% (2); mean heart dose < 25 Gy, heart V30 < 35%, and heart V40 < 25% (3); maximum spinal cord dose < 45 Gy.

### Concurrent chemotherapy

Concurrent chemotherapy regimens comprised cisplatin plus fluorouracil or paclitaxel plus cisplatin administered during thoracic radiotherapy. The cisplatin plus fluorouracil regimen involved cisplatin at 75 mg/m^2^ on day 1 and continuous intravenous fluorouracil at 1500 mg/m^2^ over 72 hours starting on day 1, repeated every 3 weeks. The paclitaxel plus cisplatin regimen was administered in two options (1): paclitaxel at 175 mg/m^2^ and cisplatin at 75 mg/m^2^ on day 1, every 3 weeks (2); paclitaxel at 50 mg/m^2^ and cisplatin at 25 mg/m^2^ on day 1, weekly.

Two levels of dose reduction were permissible for concurrent chemotherapy. Chemotherapy was discontinued in cases of grade 4 peripheral nerve toxicity, grade 2 or higher renal toxicity, or other unacceptable adverse events.

### Concurrent immune checkpoint inhibitors

Patients were administered ICIs, including tislelizumab, camrelizumab, toripalimab, and sintilimab, all of which are programmed death-1 (PD-1) inhibitors. During chemoradiotherapy, tislelizumab (200 mg), camrelizumab (200 mg), toripalimab (240 mg), and sintilimab (200 mg) were administered intravenously every 3 weeks.

Post-chemoradiotherapy, maintenance therapy with ICIs was continued every 3 weeks for up to 1 year, or until the occurrence of unacceptable toxicity, disease progression, patient refusal, or at the discretion of the treating oncologist. Dose reductions for ICIs were not permitted.

### Assessments

Laboratory tests were conducted weekly during chemoradiotherapy and every 3 weeks thereafter. Adverse events were evaluated according to the Common Terminology Criteria for Adverse Events (CTCAE, version 5.0).

Follow-up assessments were scheduled every 3 months for the first two years, every 6 months during the third to fifth year, and annually thereafter. These included physical examinations, esophagogastroduodenoscopy, contrast-enhanced cervical, chest, and abdominal computed tomography scans, electrocardiograms, echocardiography, and pulmonary function tests. Brain magnetic resonance imaging, bone scans, and 18-fluorodeoxyglucose positron emission tomography/computed tomography were performed optionally. Biopsies were conducted when recurrence was suspected.

Tumor response was independently evaluated by two experienced oncologists using the Response Evaluation Criteria in Solid Tumors (RECIST, version 1.1) post-radiotherapy completion. Treatment failures were categorized as either locoregional or distant disease, based on histological, cytological, or definitive radiological evidence. Locoregional recurrence was defined as any recurrence within the esophagus or regional lymph nodes, while distant metastasis referred to non-regional lymph node recurrence or systemic metastasis. Deaths were confirmed through official statements.

### Endpoints

The primary endpoint was progression-free survival (PFS), defined as the time from treatment initiation to disease progression or death from any cause, or censored at the last follow-up. The secondary endpoint was OS, defined as the time from treatment initiation to death, or censored at the last follow-up. Post-radiotherapy clinical response and safety were also evaluated.

### Statistical analysis

The continuous variable of age was categorized using its respective median value. The continuous variable of hemoglobin was categorized into anemia grades. The primary tumor length was categorized based on a receiver operating characteristic curve. The body mass index (BMI) was categorized into underweight (BMI < 18.5), normal weight (BMI 18.5-24.9), and overweight (BMI > 24.9). Categorical variables, including demographic factors, clinical stages, and treatment parameters, were analyzed using the χ2 test or Fisher’s exact test.

Comparisons of clinical responses post-radiotherapy and adverse events between the CCRT alone and CCRT+ICI groups were conducted. PFS and OS were compared using Kaplan-Meier methods and log-rank test statistics. Multivariable Cox regression analysis, adjusted for demographic and clinical variables, was conducted to independent prognostic factors for PFS and OS, with results presented as hazard ratios (HRs) and 95% confidence intervals (CIs).

Statistical analyses were performed using SPSS Statistics Version 26.0 software (IBM Co., Armonk, NY, USA) and R software (version 4.2.1). A two-tailed P-value of less than 0.05 was considered statistically significant in all analyses.

## Results

### Patient characteristics

**Figure 1:**
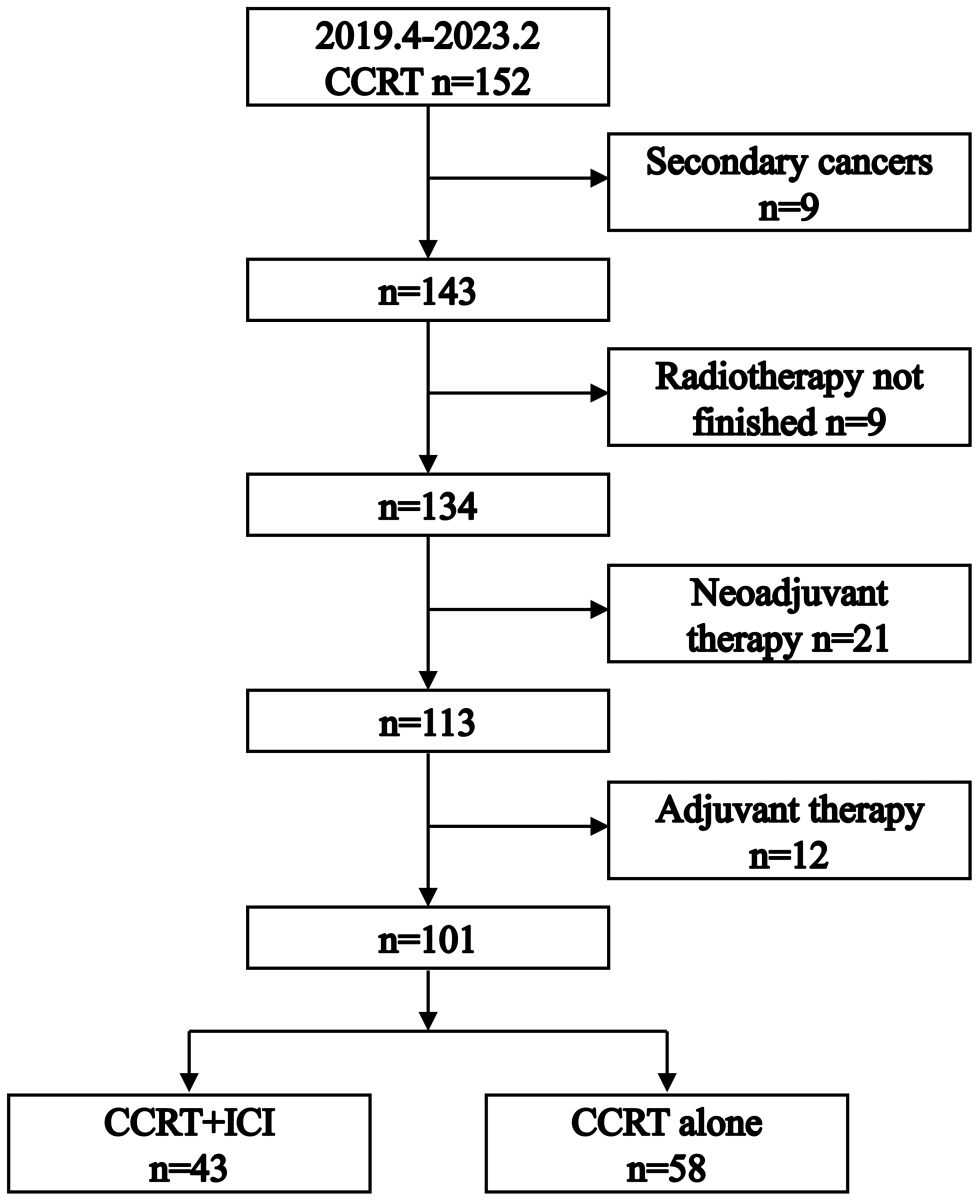
Flowchart illustrating the process of patient selection.

As illustrated in **Figure 1**, our initial cohort consisted of 152 patients who received definitive CCRT as initial treatment. After applying inclusion criteria, 101 patients were enrolled in the study: 58 in the CCRT alone group and 43 in the CCRT+ICI group. Patient characteristics are summarized in **Table 1**.

**Table 1:** Patient characteristics.

	CCRT alone (n=58)	CCRT+ICI (n=43)	P
Age (year)			0.159
≤59	35 (60.3%)	19 (44.2%)	
>59	23 (39.7%)	24 (55.8%)	
Sex			0.402
female	4 (6.9%)	6 (14.0%)	
male	54 (93.1%)	37 (86.0%)	
ECOG			0.001
0	8 (13.8%)	19 (44.2%)	
1	50 (86.2%)	24 (55.8%)	
Smoking history			0.066
no	18 (31.0%)	22 (51.2%)	
yes	40 (69.0%)	21 (48.8%)	
Alcohol history			0.009
no	14 (24.1%)	22 (51.2%)	
yes	44 (75.9%)	21 (48.8%)	
Anemia			0.994
no	46 (79.3%)	35 (81.4%)	
grade I	12 (20.7%)	8 (18.6%)	
Site			0.655
cervical	8 (13.8%)	10 (23.3%)	
upper	21 (36.2%)	14 (32.6%)	
middle	24 (41.4%)	15 (34.9%)	
distal	5 (8.6%)	4 (9.2%)	
Length (cm)			0.837
≤5.55	28 (48.3%)	19 (44.2%)	
>5.55	30 (51.7%)	24 (55.8%)	
BMI (kg/m^2^)			0.209
18.5-24.9	37 (63.8%)	27 (62.8%)	
<18.5	14 (24.1%)	6 (14.0%)	
>24.9	7 (12.1%)	10 (23.2%)	
T stage			0.327
T2	5 (8.6%)	1 (2.3%)	
T3	37 (63.8%)	32 (74.4%)	
T4	16 (27.6%)	10 (23.3%)	
N stage			0.203
N0	3 (5.2%)	6 (14.0%)	
N1	21 (36.2%)	20 (46.5%)	
N2	25 (43.1%)	12 (27.9%)	
N3	9 (15.5%)	5 (11.6%)	
AJCC stage			0.263
stage II	2 (3.5%)	5 (11.6%)	
stage III	38 (65.5%)	27 (62.8%)	
stage IVa	18 (31.0%)	11 (25.6%)	
Radiotherapy dose			<0.001
60Gy	39 (67.2%)	42 (97.7%)	
50.4Gy	19 (32.8%)	1 (2.3%)	
Chemotherapy regimen			0.088
TP	42 (72.4%)	38 (88.4%)	
PF	16 (27.6%)	5 (11.6%)	

CCRT, concurrent chemoradiotherapy; ICI, immune checkpoint inhibitors; ECOG, Eastern Cooperative Oncology Group; Hgb, haemoglobin; MBI, body mass index; AJCC, American Joint Committee on Cancer; TP, paclitaxel plus cisplatin; PF, cisplatin plus fluorouracil.

Baseline clinical characteristics, such as age, sex, smoking history, anemia grade, tumor site, primary tumor length, BMI, clinical T stage, clinical N stage, chemotherapy regimen, staging according to the eighth edition of the American Joint Committee on Cancer, and chemotherapy regimen, were comparably distributed between the two groups. The CCRT alone group had a higher proportion of patients with an Eastern Cooperative Oncology Group performance status score of 1 (86.2%) and a history of alcohol use (75.9%). Conversely, a greater percentage of patients in the CCRT+ICI group received a radiotherapy dose of 60 Gy (97.7%).

The median follow-up time was 21.5 months (interquartile range [IQR]: 12-27 months) for the CCRT alone group and 9 months (IQR: 5.5-14.5 months) for the CCRT+ICI group, with the last follow-up conducted in August 2023. Four patients were lost to follow-up: three in the CCRT alone group and one in the CCRT+ICI group.

### Treatment patterns

The majority of patients in both groups received the paclitaxel plus cisplatin regimen for concurrent chemotherapy (72.4% in the CCRT alone group and 88.4% in the CCRT+ICI group). The cisplatin plus fluorouracil regimen was administered to 27.6% of patients in the CCRT alone group and 11.6% in the CCRT+ICI group. The median number of concurrent chemotherapy cycles was 2 (IQR: 1-3 cycles) in the CCRT alone group and 3 (IQR: 2-4.5 cycles) in the CCRT+ICI group.

In the CCRT+ICI group, 10 (23.2%) patients received 1 cycle, 11 (25.6%) received 2 cycles, and 22 (51.2%) received 3 cycles of concurrent immunotherapy. Post-radiotherapy, 32 patients were under observation, primarily due to patient refusal (n=11) or investigator decision (n=21). For maintenance therapy, 11 (25.6%) patients received ICIs, with a median of 1 cycle (IQR: 1-3 cycles). Discontinuation of maintenance immunotherapy was due to disease progression (n=1), adverse events (n=5), patient refusal (n=3), or investigator decision (n=1).

### Response after completion of radiotherapy

Clinical response evaluation post-radiotherapy was feasible for 90 (89.1%) patients (**Table 2**). In the CCRT alone group, 1 (1.7%) patient achieved a complete response, compared to 5 (11.6%) patients in the CCRT+ICI group (P = 0.037). Partial response rates were 44.8% in the CCRT alone group and 41.9% in the CCRT+ICI group. The objective response rates were 46.5% and 53.5% for the CCRT alone and CCRT+ICI groups, respectively. Non-evaluation of response post-radiotherapy was primarily due to patient refusal.

**Table 2:** Clinical response after completion of radiotherapy.

	CCRT alone (n=58)	CCRT+ICI (n=43)	P
Complete response			0.037
no	57 (98.3%)	38 (88.4%)	
yes	1 (1.7%)	5 (11.6%)	
Partial response			0.766
no	32 (55.2%)	25 (58.1%)	
yes	26 (44.8%)	18 (41.9%)	
Stable disease			0.097
no	31 (53.4%)	30 (69.8%)	
yes	27 (46.6%)	13 (30.2%)	
Progressive disease			0.999
no	58 (100.0%)	43 (100.0%)	
yes	0 (0.0%)	0 (0.0%)	
Unknown			0.134
no	54 (93.1%)	36 (83.7%)	
yes	4 (6.9%)	7 (16.3%)	
Objective response rate			0.491
no	31 (53.4%)	20 (46.5%)	
yes	27 (46.6%)	23 (53.5%)	
Disease control rate			0.134
no	4 (6.9%)	7 (16.3%)	
yes	54 (93.1%)	36 (83.7%)	

CCRT, concurrent chemoradiotherapy; ICI, immune checkpoint inhibitors.

### Progression-free survival

**Figure 2:**
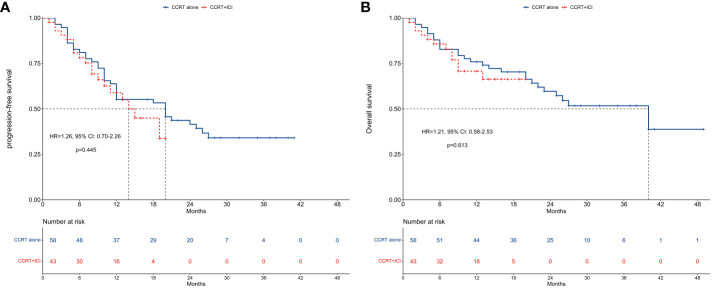
Survival between concurrent chemoradiotherapy and concurrent chemoradiotherapy plus immune checkpoint inhibitors. **(A)** Progression-free survival. **(B)** Overall survival.

Kaplan-Meier curves and risk tables for PFS are depicted in **Figure 2A**. The median PFS was 20 months for the CCRT alone group and 14 months for the CCRT+ICI group, with 1-year PFS rates of 55.2% and 58.9%, respectively.

**Figure 3:**
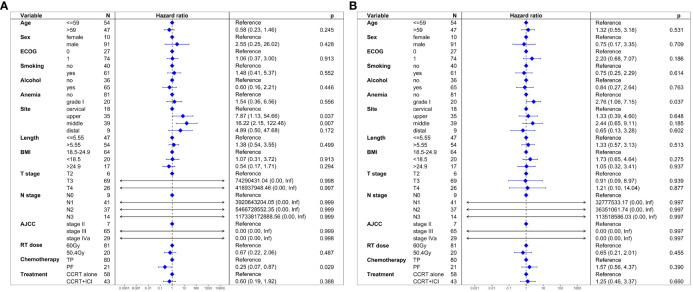
Multivariate regression analysis of prognostic factors. **(A)** Progression-free survival. **(B)** Overall survival.

In unadjusted analysis, no significant difference in PFS was observed between the two groups (HR = 1.26, 95% CI: 0.70-2.26; P = 0.445). Multivariable proportional hazard regression analysis also indicated that CCRT+ICI was not an independent prognostic factor for PFS (HR = 0.60, 95% CI: 0.19-1.92; P = 0.388; **Figure 3A**).

### Overall survival

**Figure 2B** presents Kaplan-Meier curves and risk tables for OS. The median OS for the CCRT alone group was 40 months, whereas it was not reached in the CCRT+ICI group. The 1-year OS rates were 75.9% for the CCRT alone group and 70.8% for the CCRT+ICI group.

Similar to PFS, unadjusted analysis revealed no significant difference in OS between the groups (HR = 1.21, 95% CI: 0.58-2.53; P = 0.613). Multivariable analysis confirmed that CCRT+ICI was not an independent prognostic factor for OS (HR = 1.25, 95% CI: 0.46-3.37; P = 0.660; **Figure 3B**).

### Survivals among different concurrent immunotherapy cycles

**Figure 4:**
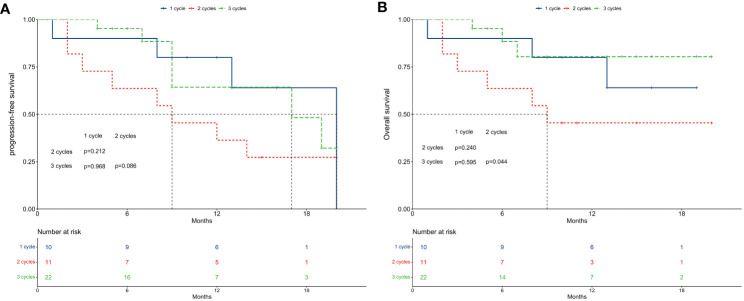
Survival between subgroups of concurrent immune checkpoint inhibitors cycles. **(A)** Progression-free survival. **(B)** Overall survival.

**Figures 4A, B** illustrate PFS and OS across different concurrent immunotherapy cycles. For PFS, median rates were 20 months for 1 cycle, 9 months for 2 cycles, and 17 months for 3 cycles. The 1-year PFS rates were 80.0% for 1 cycle, 36.4% for 2 cycles, and 64.3% for 3 cycles. No significant differences in PFS were observed across the different immunotherapy cycles (P > 0.05 in pairwise comparisons).

Regarding OS, median rates were not reached for 1 cycle, 9 months for 2 cycles, and not reached for 3 cycles. The 1-year OS rates were 80.0% for 1 cycle, 45.5% for 2 cycles, and 80.4% for 3 cycles. While no significant differences were found between 1 cycle and 2 cycles (P = 0.240), or 1 cycle and 3 cycles (P = 0.595), patients receiving 2 cycles exhibited significantly worse OS compared to those receiving 3 cycles (P = 0.044).

### Adverse events

Adverse events are detailed in **Table 3**. The CCRT alone group exhibited a higher incidence of leukopenia of any grade (93.1% vs. 76.7%), whereas the CCRT+ICI group had a higher incidence of pneumonitis of any grade (36.2% vs. 65.1%). The incidence of adverse events of grade 3 or higher was comparable between the two groups.

**Table 3:** Adverse events.

	Any grade			Grade ≥3		
	CCRT alone (n=58)	CCRT+ICI (n=43)	P	CCRT alone (n=58)	CCRT+ICI (n=43)	P
Hematological events						
Leukopenia			0.039			0.990
no	4 (6.9%)	10 (23.3%)		35 (60.3%)	26 (60.5%)	
yes	54 (93.1%)	33 (76.7%)		23 (39.7%)	17 (39.5%)	
Neutropenia			0.213			0.843
no	15 (25.9%)	17 (39.5%)		44 (75.9%)	31 (72.1%)	
yes	43 (74.1%)	26 (60.5%)		14 (24.1%)	12 (27.9%)	
Anemia			0.983			0.870
no	8 (13.8%)	5 (11.6%)		53 (91.4%)	38 (88.4%)	
yes	50 (86.2%)	38 (88.4%)		5 (8.6%)	5 (11.6%)	
Thrombocytopenia			0.150			0.999
no	34 (58.6%)	32 (74.4%)		53 (91.4%)	40 (93.0%)	
yes	24 (41.4%)	11 (25.6%)		5 (8.6%)	3 (7.0%)	
Non-hematological events						
Liver dysfunction			0.999			0.999
no	53 (91.4%)	39 (90.7%)		57 (98.3%)	43 (100.0%)	
yes	5 (8.6%)	4 (9.3%)		1 (1.7%)	0 (0.0%)	
Creatine kinase increased			0.243			0.243
no	58 (100.0%)	42 (97.7%)		58 (100.0%)	42 (97.7%)	
yes	0 (0.0%)	1 (2.3%)		0 (0.0%)	1 (2.3%)	
Hypothyroidism			0.349			0.880
no	58 (100.0%)	41 (95.3%)		58 (100.0%)	42 (97.7%)	
yes	0 (0.0%)	2 (4.7%)		0 (0.0%)	1 (2.3%)	
Nausea			0.182			0.998
no	41 (70.7%)	24 (55.8%)		57 (98.3%)	43 (100.0%)	
yes	17 (29.3%)	19 (44.2%)		1 (1.7%)	0 (0.0%)	
Vomiting			0.470			0.612
no	47 (81.0%)	38 (88.4%)		56 (96.6%)	43 (100.0%)	
yes	11 (19.0%)	5 (11.6%)		2 (3.4%)	0 (0.0%)	
Diarrhea			0.998			0.998
no	57 (98.3%)	42 (97.7%)		57 (98.3%)	43 (100.0%)	
yes	1 (1.7%)	1 (2.3%)		1 (1.7%)	0 (0.0%)	
Dermatitis			0.349			0.999
no	58 (100.0%)	41 (95.3%)		58 (100.0%)	43 (100.0%)	
yes	0 (0.0%)	2 (4.7%)		0 (0.0%)	0 (0.0%)	
Fistula			0.999			0.999
no	56 (96.6%)	41 (95.3%)		56 (96.6%)	41 (95.3%)	
yes	2 (3.4%)	2 (4.7%)		2 (3.4%)	2 (4.7%)	
Pneumonitis			0.008			0.421
no	37 (63.8%)	15 (34.9%)		56 (96.6%)	39 (90.7%)	
yes	21 (36.2%)	28 (65.1%)		2 (3.4%)	4 (9.3%)	

CCRT, concurrent chemoradiotherapy; ICI, immune checkpoint inhibitors.

## Discussion

In the evolving landscape of esophageal cancer treatment, ICIs have emerged as significant therapeutic agents. However, our retrospective cohort study suggests that while the combination of CCRT with ICIs led to an improved complete response rate compared to CCRT alone in patients with locally advanced esophageal squamous cell carcinoma, this did not translate into enhanced survival outcomes for this patient cohort.

Previous studies have reported encouraging pathologic complete response rates following neoadjuvant chemoradiotherapy combined with ICIs, with rates of 25% for adenocarcinoma and 55.6% for squamous cell carcinoma (16, 17). In studies where patients received CCRT combined with ICIs, complete response rates varied, ranging from 10% at a radiation dose of 40 Gy to 62% three months post-radiotherapy (18, 19). In contrast, our study observed a complete response rate of 11.6%, aligning more closely with the lower end of this spectrum, specifically comparable to the rate observed at 40 Gy radiation but notably lower than the rate at 3 months post-radiotherapy.

This variance in complete response rates could be attributed to several factors. A primary consideration is the timing of response assessment in our study, which was conducted immediately after the completion of radiotherapy. This approach may not have fully captured the extent of response that could develop over time. Additionally, the lack of response evaluations using 18-fluorodeoxyglucose positron emission tomography/computed tomography at the time of assessment in our study might have contributed to the relatively lower observed complete response rate. Such imaging techniques can provide more comprehensive insights into metabolic activity and response to treatment of the tumor, potentially revealing a more significant response than what is observable through standard assessment methods alone.

In evaluating treatment outcomes, previous researches have highlighted favorable 1-year PFS and OS rates when ICIs were combined with CCRT (18, 19). Specifically, the combination of camrelizumab with CCRT achieved a 1-year PFS rate of 80.0% (18), and toripalimab with CCRT resulted in a 1-year PFS rate of 54.5% (19). Similarly, the 1-year OS rates were reported as 85.0% for camrelizumab plus CCRT and 78.4% for toripalimab plus CCRT (18, 19).

In our study, the observed 1-year PFS rate was 58.9%, which aligns more closely with the outcome for toripalimab but is lower than that for camrelizumab. More notably, the 1-year OS rate in our study was 70.8%, which is lower than the rates observed with both camrelizumab and toripalimab. This disparity in survival outcomes, particularly in the context of OS, may contribute to understanding the lack of significant differences observed between the CCRT+ICI and CCRT alone groups in our research.

Several factors might have contributed to the outcomes observed in our study. A notable consideration is the diversity of ICIs utilized, encompassing tislelizumab (37.2%), camrelizumab (39.6%), toripalimab (16.3%), and sintilimab (6.9%). All these agents are PD-1 inhibitors. The use of a variety of ICIs could have potentially impacted the treatment outcomes. Despite this diversity, our findings suggest that, as a collective group, PD-1 inhibitors did not significantly enhance survival rates in our patient cohort.

Another factor warranting consideration is the number of concurrent immunotherapy cycles administered. Intriguingly, our data indicated that patients who received two cycles of concurrent immunotherapy exhibited worse OS compared to those who received either one or three cycles. This observation suggests a complex relationship between the number of immunotherapy cycles and patient outcomes.

One hypothesis is that patients receiving only one cycle of concurrent immunotherapy might have experienced adverse events related to the treatment, leading to the discontinuation of therapy. Interestingly, the occurrence of immunotherapy-induced adverse events has been suggested as a positive prognostic factor for OS in some studies (22, 23). This counterintuitive finding might reflect a more robust immune response in these patients, potentially translating into better survival outcomes.

Conversely, receiving three cycles of concurrent immunotherapy could confer a stronger anti-cancer effect compared to just two cycles. This might be due to a more sustained engagement of the immune system with the tumor, leading to improved control of the disease. However, the exact mechanisms behind this potential dose-response relationship remain unclear and warrant further investigation.

To the best of our knowledge, this retrospective cohort study is the first to provide real-world treatment outcomes comparing CCRT combined with ICIs against CCRT alone in patients with locally advanced esophageal squamous cell carcinoma. Our findings highlight the potential drawbacks of integrating ICIs with CCRT in this specific patient population. However, several limitations of our study must be acknowledged.

Firstly, the relatively small sample size of our study may have constrained its statistical power, which is the ability to detect a true effect or difference when it exists. Consequently, our study might be underpowered to identify subtle yet clinically significant differences or effects. Despite this limitation, we utilized multivariable Cox regression analysis, adjusting for demographic factors, clinical stages, and treatment parameters, to identify independent prognostic factors for both PFS and OS. While CCRT+ICI did not emerge as an independent prognostic factor, our findings should be interpreted as preliminary insights rather than definitive conclusions.

Secondly, the median follow-up time of 9 months (IQR: 5.5-14.5 months) for the CCRT+ICI group may not adequately capture the full impact of the treatment on OS, especially considering the potential for long-term benefits associated with immunotherapies. Extended follow-up periods could provide more comprehensive data on the durability and longevity of treatment effects. With longer-term data, differences between the treatment groups might become more pronounced, revealing late recurrences or delayed treatment benefits not observable within shorter follow-up periods. Future studies with extended follow-up are necessary to fully understand these long-term outcomes.

Finally, conducted at a single hospital, treatment decisions of our study were made by clinicians in a real-world clinical context, potentially introducing unmeasured confounding factors. While this approach reflects actual clinical practice, it adds complexity to the analysis of treatment outcomes. To overcome these limitations and validate our findings, results from ongoing clinical trials, such as RATIONALE 311 and ESCORT-CRT, will be crucial.

In conclusion, our study provides preliminary evidence suggesting that the combination of CCRT with ICIs may not confer improved survival outcomes compared to CCRT alone in patients with locally advanced esophageal squamous cell carcinoma. This observation, drawn from our single-center cohort with its specific sample size, highlights the complexity of treating this disease and the need for cautious interpretation of these results.

## Data availability statement

The raw data supporting the conclusions of this article will be made available by the authors, without undue reservation.

## Ethics statement

Ethics approval was waived by the ethics committee/Institutional Review Board of Guangxi Medical University Cancer Hospital due to its retrospective cohort design. The studies were conducted in accordance with the local legislation and institutional requirements. The ethics committee/institutional review board also waived the requirement of written informed consent for participation from the participants or the participants’ legal guardians/next of kin because Ethics approval was waived by the ethics committee/Institutional Review Board of Guangxi Medical University Cancer Hospital due to its retrospective cohort design.

## Author contributions

J-QH: Conceptualization, Writing – original draft. H-WL: Methodology, Writing – original draft. YL: Methodology, Writing – original draft. LC: Resources, Writing – original draft. SP: Resources, Writing – original draft. B-BY: Validation, Writing – original draft. X-BP: Conceptualization, Writing – review & editing.
